# Pharmacological Conditioning of the Heart: An Update on Experimental Developments and Clinical Implications

**DOI:** 10.3390/ijms22052519

**Published:** 2021-03-03

**Authors:** Sebastian Roth, Carolin Torregroza, Katharina Feige, Benedikt Preckel, Markus W. Hollmann, Nina C. Weber, Ragnar Huhn

**Affiliations:** 1 Department of Anesthesiology, Medical Faculty and University Hospital Duesseldorf, Heinrich-Heine-University Duesseldorf, Moorenstr. 5, 40225 Duesseldorf, Germany; sebastian.roth@med.uni-duesseldorf.de (S.R.); katharinakristina.feige@med.uni-duesseldorf.de (K.F.); ragnar.huhn@med.uni-duesseldorf.de (R.H.); 2 Department of Anesthesiology, Amsterdam University Medical Center (AUMC), Location AMC, Meibergdreef 9, 1100 DD Amsterdam, The Netherlands; b.preckel@amsterdamumc.nl (B.P.); m.w.hollmann@amsterdamumc.nl (M.W.H.); n.c.hauck@amsterdamumc.nl (N.C.W.)

**Keywords:** cardioprotection, molecular mechanisms, preconditioning, postconditioning, ischemia reperfusion injury

## Abstract

The aim of pharmacological conditioning is to protect the heart against myocardial ischemia-reperfusion (I/R) injury and its consequences. There is extensive literature that reports a multitude of different cardioprotective signaling molecules and mechanisms in diverse experimental protocols. Several pharmacological agents have been evaluated in terms of myocardial I/R injury. While results from experimental studies are immensely encouraging, translation into the clinical setting remains unsatisfactory. This narrative review wants to focus on two aspects: (1) give a comprehensive update on new developments of pharmacological conditioning in the experimental setting concentrating on recent literature of the last two years and (2) briefly summarize clinical evidence of these cardioprotective substances in the perioperative setting highlighting their clinical implications. By directly opposing each pharmacological agent regarding its recent experimental knowledge and most important available clinical data, a clear overview is given demonstrating the remaining gap between basic research and clinical practice. Finally, future perspectives are given on how we might overcome the limited translatability in the field of pharmacological conditioning.

## 1. Introduction

**Figure 1:**
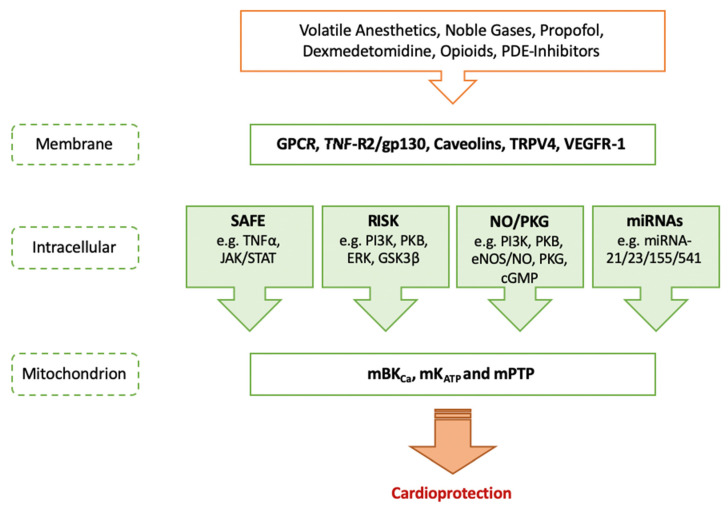
Molecular targets of pharmacological conditioning. GPCR = G protein-coupled receptor; TNF-R2 = tumor necrosis factor receptor 2; gp130 = glycoprotein 130; TRPV4 = transient receptor potential vanilloid 4; VEGFR-1 = vascular endothelial growth factor receptor 1; SAFE = survivor activating factor enhancement; TNFα = tumor necrosis factor alpha; JAK/STAT = janus kinase/signal transducers and activators of transcription; RISK = reperfusion injury salvage kinase; PI3K = phosphatidylinositol 3-kinase; PKB = protein kinase B; ERK = extracellular-signal-regulated kinase; GSK3β = glycogen synthase kinase 3 beta; NO/PKG = nitric oxide/protein kinase G; eNOS = endothelial nitric oxide synthase; cGMP = cyclic guanosine monophosphate; miRNA = micro ribonucleic acid; mBK_Ca_ = mitochondrial large-conductance calcium-sensitive potassium channel, mK_ATP_ = mitochondrial adenosine triphosphate-sensitive potassium channel; mPTP = mitochondrial permeability transition pore.

Conditioning is still the strongest cardioprotective mechanism to reduce ischemia-reperfusion (I/R) injury and cell death after myocardial infarction (MI). In 1986, Murry et al. were the first to describe that short periods of non-lethal coronary artery occlusions followed by reperfusion reduced cardiac infarct size after global ischemia [1]. This phenomenon was called ischemic preconditioning (IPC). However, due to its invasiveness, it is rather impractical for the clinical setting. Another way to protect the heart against the consequences of I/R injury is pharmacological conditioning, a concept that is based on the administration of specific drugs mimicking the effect of IPC. Several pharmacological agents have been evaluated regarding protection against myocardial I/R injury, e.g., volatile anesthetics, opioids or alpha-2 agonists, and further a multitude of different signaling molecules and mechanisms of conditioning have been identified in the experimental setting (as shown in Figure 1). The results from experimental studies are encouraging. However, clinical trials on different conditioning strategies have revealed inconclusive, neutral or even negative results. This review will address the significant gap between new developments of pharmacological conditioning in the experimental setting and current clinical implications of the respective agents in more detail. Finally, future perspectives on how to possibly overcome the limited translatability in the field of pharmacological conditioning are illustrated. With regard to the exact pathways of cardioprotection, we refer to previously published articles where general underlying mechanisms are explained more in depth [2,3]. More detailed information on current clinical evidence and potential reasons for the limited translation can be found in further review articles [3,4]. In the following, most important substances for pharmacological conditioning of the heart in the perioperative setting are presented systematically regarding experimental developments and clinical implications of each agent.

## 2. Volatile Anesthetics

### 2.1. Experimental Developments

To date, there are three routinely used volatile anesthetics that have been investigated in the context of pharmacological conditioning: sevoflurane, isoflurane and desflurane.

Sevoflurane confers cardioprotection through pre- and post-conditioning [5,6] via several key pathways as well as mitochondrial adenosine triphosphate-sensitive potassium channel (mK_ATP_) activation and modulation of mitochondrial permeability transition pore (mPTP) (Figure 1) [2,7]. In addition, apoptosis is modulated by sevoflurane-induced postconditioning via the janus kinase-signal transducer and activator of transcription 3 (JAK/STAT3) pathway. A JAK2 selective inhibitor fully abrogates cardioprotection by sevoflurane in a rat model of myocardial I/R injury [8]. In addition, a pivotal role for autophagy in cardiac conditioning with sevoflurane has been described [9]. A study by Qiao et al. underlined these findings in an in vivo and in vitro rat model showing that sevoflurane-induced postconditioning confers cardioprotection. Administration of sevoflurane improves autophagic flux by a nitric oxide (NO)-dependent mechanism [10]. The beneficial effects of sevoflurane were fully abolished by administration of the nitric oxide synthase (NOS) inhibitor N omega-nitro-L-arginine methyl ester (L-NAME) as well as by the autophagic flux blocker chloroquine (CQ) [10].

Next to the commonly known pathways of cardioprotection, in the last two years research mainly focused on the identification of different cell types as cardioprotective molecular targets. Vascular endothelial growth factor receptor (VEGFR)—more specifically VEGFR-1—is one of these targets. Qian et al. demonstrated that treatment with sevoflurane results in an increase of VEGFR-1 expression along with a decrease in markers of inflammation [11]. This remained effective when adding a selective VEGFR-1 agonist (placenta growth factor (PlGF)), while administration of a specific VEGFR-1 blocker (macrophage migration inhibitory factor-1 (MIF-1)) completely abolished protection [11]. Another aspect recent research activity focused on refers to the role of non-coding ribonucleic acids (RNA) (Figure 1). Referring to microRNAs (miRNA) and conditioning strategies, sevoflurane-induced cardioprotection seems to be regulated by small RNAs [12], for example by a decrease of miRNA-155 [13]. In turn, sirtuin1 (SIRT1), a target gene of the respective miRNA, is increased and leads to inhibition of cardiomyocyte apoptosis.

Fortunately, preconditioning with sevoflurane is not negatively affected by diabetic conditions, which is a promising approach regarding successful translation into the clinical setting. In this context, Xie et al. showed that treatment with sevoflurane protects the heart via AMP-activated protein kinase (AMPK)-dependent inhibition of pro-death mitogen-activated protein kinase p38 (p38 MAPK) in non-diabetic mice [14]. In contrast, the volatile anesthetic confers its cardioprotective effects in diabetic animals completely independent of AMPK [14]. While these experimental results on diabetes and sevoflurane-induced cardioprotection are promising for the clinical context, unfortunately the beneficial effects of sevoflurane are abrogated in aged hearts. Studies indicate that in this context, sevoflurane is possibly influenced by a changed modulation in genes regulated by the nuclear transcription factor kappa B (NFkB) [15].

The second routinely used volatile anesthetic is isoflurane. First investigations on its cardioprotective effects were performed in the 1980s: Warltier et al. demonstrated that inhalation of isoflurane results in a significantly reduced infarct size in stunned canine myocardium [16]. Numerous studies followed and investigated isoflurane-induced pre- and post-conditioning [17,18,19]. The majority of experimental studies was conducted in young and healthy animals. However, there is an aging population with increasing co-morbidities and co-medications. Therefore, recent research activities focused on the impact of aging. One compound that has been suggested in this context is the potent antioxidant TEMPOL reported to avoid the inhibitory effects of aging on isoflurane-induced cardioprotection in rats by improving mitochondrial function. In contrast, inhibition of autophagy and mitophagy abolished protection mediated by isoflurane in cardiomyocytes from both young and TEMPOL pretreated old rats [20]. In general, there are controversies regarding the role of mitophagy and autophagy in cardioprotective strategies [21]. Mitophagy prevents potential detrimental reactive oxygen species (ROS) production in damaged mitochondria after I/R, is involved in cardiac preconditioning and declines with age. Therefore, mitophagy might be a crucial target of cardioprotection in the elderly heart [22]. Cheng et al. showed that treatment with isoflurane reduced autophagy and increased cell viability of primary cardiomyocytes and thus protection against anoxia/reoxygenation [23]. This effect was mediated by a reduced expression of nucleotide binding oligomerization domain containing 2 (NOD2), which is usually accompanied with higher expression of autophagy-related genes and increased phosphorylation of p38 MAPK [23]. A variety of previous studies have demonstrated cardioprotective effects against I/R injury by increased autophagy; however, excessive autophagy plays a pivotal role in the reperfusion-induced damage on cardiac function (as reviewed in [21]). These controversial findings are just examples emphasizing the importance of further unravelling the basic mechanisms of cardioprotection. Moreover, the central role of mitochondria in conditioning strategies is highlighted and reflects why most recent research has mainly focused on this aspect. For example, Xu et al. demonstrated that treatment with isoflurane in isolated, myocardial mitochondria results in an uncoupling effect during state 2 respiration and attenuates state 3 respiration independent of endogenous mitochondrial NO [24]. Alongside this work, Harisseh and coworkers investigated effects of different anesthetics on mitochondrial activity dependent on Cyclophilin D (CypD)—a main mediator of mitochondrial dysfunction and component of the mPTP [25]. CypD plays a major role in regulation of mitochondria by regulating mPTP, e.g., through decreasing the threshold for opening of the mPTP triggered by phosphate or calcium (Ca^2+^) [26]. Isoflurane inhibits state 3 respiration in complex I of mitochondria, decreases membrane potential and enhances adenosine diphosphate (ADP) consumption duration in wildtype (WT) as well as CypD knockout mice [25].

Previous studies revealed that isoflurane confers cardioprotective effects in mouse hearts against I/R injury through a miRNA-21-dependent mechanism [27,28]. MiRNA-21, which is highly expressed in cardiomyocytes and upregulated by isoflurane, exerts effects on different cardioprotective signaling pathways, like phosphorylation of protein kinase B (PKB) or endothelial NOS (eNOS). More recently, Liu et al. demonstrated that treatment with isoflurane also reduces expression of miRNA-23 in cardiomyocytes resulting in increased resistance against oxidative stress [29]. Consistently, overexpressing of miRNA-23 seems to sensitize cardiomyocytes to oxidative stress. These findings clarify that protection of cardiomyocytes against I/R injury by isoflurane might be mediated by suppression of miRNA-23 [29].

Finally, the third routinely used volatile anesthetic is desflurane. To date, experimental data on its cardioprotective properties are scarce. Interestingly, Heiberg et al. reported that the protective effect of desflurane is superior to that of propofol. However, a combination of both agents fully abolished cardioprotective effects [30]. Further studies are needed regarding desflurane and its effectiveness in pharmacological cardiac conditioning.

### 2.2. Clinical Implications

With regard to the clinical setting, there is no final answer regarding cardioprotective effects of volatile anesthetics (Table 1). In a meta-analysis Uhlig et al. reported that anesthesia with volatile anesthetics in cardiac surgery may reduce mortality [31]. However, no benefits were seen in non-cardiac surgical patients. In a moderate-sized randomized controlled trial (RCT) including 868 patients undergoing coronary artery bypass graft (CABG) surgery, a reduced length of hospital stay and a reduction in one-year mortality was observed when using sevoflurane instead of total intravenous anesthesia employing propofol [32]. In contrast, another trial (*n* = 200) did not reveal any beneficial in terms of prolonged intensive care unit stay, mortality, or both in patients undergoing high-risk cardiac surgery [33]. Most robust data come from a pragmatic, multicenter RCT by Landoni and colleagues including 5400 patients undergoing isolated CABG surgery [34]. This study compared the use of volatile anesthetics (desflurane, sevoflurane, isoflurane) at any given moment during anesthesia versus propofol-based total intravenous anesthesia and found no significant reduction in mortality one year after surgery [34]. There were also no significant differences regarding secondary outcomes such as myocardial infarction or other adverse events [34]. In conclusion—and as recommended by current guidelines—volatile anesthetics may be preferable especially in cardiac surgery patients, but definite evidence is lacking.

## 3. Helium—A Noble Gas

### 3.1. Experimental Developments

Several aforementioned mechanisms have been described for another type of inhalational drugs: noble gases (e.g., xenon, helium, neon and argon). These gases are supposed to be chemical ‘inert’, meaning a lack of chemical reactivity. However, they have proven to be far away from being biological inert. They exert a pronounced cardioprotective effect that has not only been described in vitro and in vivo in animals, but also in humans [39].

Since noble gases are monoatomic gases with a filled outer shell of valence electrons an interaction with other compounds was suggested highly unlikely. Hence, findings indicating cardioprotection by noble gases in vivo were quite surprising [40]. Helium, argon and neon—compared to xenon—do not confer a hypnotic effect, thus its cardioprotective effect must be separate from any anesthetic properties of the noble gases. This makes helium an interesting candidate for an easily applicable intervention as it can be given to awake patients experiencing an I/R situation. Moreover, helium is already available for clinical use in patients suffering from respiratory diseases.

Referring to comprehensive reviews of several mechanisms involved in helium induced cardioprotection [39,41,42,43], we want to focus on one key mechanism conferring the effects of helium within the cell to the target proteins ultimately leading to cardioprotection. The determination of caveolins as activators of a multi protein signaling pathway connecting the cell membrane to downstream targets, has been the latest steps fully elucidating the cardioprotective mechanisms of an ‘inert’ gas like helium. Caveolins are small proteins that are anchored into so called caveolae in the plasma membrane acting as structural components of the cellular membrane [44,45,46,47]. Caveolins partly build caveolae and their scaffolding domain has a key role in binding proteins that have been described to be involved in helium conditioning of the heart: e.g., the G-alpha subunit of heterotrimeric G-proteins, Src kinases, phosphatidylinositol 3-kinase (PI3K), eNOS, protein kinase C (PKC) isoforms and extracellular-signal-regulated kinase (ERK) [48,49,50]. Furthermore, these small proteins mediate several responses in stress adaptation processes [51]. The isoforms caveolin 1 and 2 are expressed in a variety of cell types, e.g., endothelial cells [52]. In contrast, caveolin 3 is predominantly found in skeletal, cardiac muscle and certain smooth muscle cells [53]. The properties of caveolae and caveolins in transmitting a signal from the cell membrane into the cell itself make them a very promising molecular target for noble gases like helium. Additionally, for the inhalational anesthetic isoflurane there is convincing evidence that caveolins are also a key mediator in the cardioprotection exerted by the volatile anesthetic [52,54].

Recent in vivo studies have shown that caveolin 1 and 3 can be identified as key mediators of helium induced cardioprotection. In a rat model of I/R injury, helium used as inductor of postconditioning increases caveolin 3 levels in plasma of the animals. Interestingly,—in contrast to the non-infarcted area—in the infarcted myocardium both caveolin 1 and 3 levels were increased [55]. In line with these findings, in a study of cardiac arrest and subsequent resuscitation in rats, application of helium for five minutes as preconditioning stimulus also revealed differential regulation of both proteins within the myocardium accompanied by a reduced cardiac apoptosis [56]. In contrast, in a study of helium-induced preconditioning in isolated Langendorff perfused mice hearts no protection was found [57]. Given that the Langendorff model lacks any blood circulation, further studies were carried out assessing the distribution and expression of caveolins in mice hearts in more detail. In fact, a decrease in caveolin 1 and 3 expression in the membrane fractions of hearts was observed [57]. This effect was accompanied by an increase of both proteins in the platelet free plasma of mice [57]. Thus, the missing blood component in the respective Langendorff model might be one of the carriers of the protective caveolin. Helium is able to induce the secretion of caveolin into the blood stream, and thereby, the protective factors are transported to the target organ. These findings were supported by in vitro experiments showing increased mitochondrial metabolism of muscle cells that were treated with serum from mice after inhalation of helium [57].

### 3.2. Clinical Implications

Although helium might seem to be an ideal candidate for drug-induced cardioprotection, there is yet no compelling evidence proofing that helium administration indeed protects patients at risk for an ischemic insult (Table 1). Thus, all the above described promising experimental results are yet to be translated into the clinical scenario.

In contrary, limited clinical data suggest no protection in patients undergoing CABG [58]. 125 CABG patients were included in a prospective, placebo controlled, investigator blinded, parallel arm single-center study. Patients randomly assigned to ventilation with helium (70% in a gas mixture) for three x five minutes before the start of the cardiopulmonary bypass, after the release of the cross clamp (referring to helium pre- and postconditioning) or in a combination of both strategies. However, none of the applied conditioning protocols had a protective effect on post-operative troponin release [58]. Furthermore, neither group showed an upregulation of potential molecular targets within the human heart—p38 MAPK, ERK 1/2 or levels of heat shock protein 27 (HSP27) and PKC-ε—indicating no involvement of these targets [58].

Interestingly, there are a few small studies in humans assessing inflammation related effects of helium on the vascular system rather than direct cardioprotective effects. However, results from these studies might also contribute to understanding the cardioprotective mechanisms of helium, considering a compromised vascular system also contributes to cardiac dysfunction. In a forearm blood flow model of I/R in healthy volunteers, results showed that three times of five minutes 79% helium inhalation improved post-ischemic endothelial dysfunction and protection was still effective even up to 24 h after helium inhalation [59]. Unfortunately, potentially involved molecular targets, like cytokines, adhesion molecules, or microparticles, were not influenced by helium in this study [59]. In contrast, a study addressing post-ischemic reactive hyperemia by administering 50% helium before, during and after forearm ischemia failed to show endothelial protection [60]. In fact, only a modest anti-inflammatory activity of helium—shown in a decrease of intracellular adhesion molecule (CD) 1 and CD11b on leukocytes as well as a reduced expression of pro-coagulant markers CD42b and P-selectin glycoprotein ligand-1 on platelets—was detected [60].

Taken together, even though animal and cell experimental studies showed promising cardioprotective effects, translation of helium as a cardioprotective strategy for clinical use has yet failed and more large randomized clinical trials are needed.

## 4. Propofol

### 4.1. Experimental Developments

Due to its contradictory behavior, propofol takes a special role in the field of pharmacological conditioning: on the one hand it is suggested to provide cardioprotection itself, but on the other hand it seems to negatively affect cardioprotective properties of other substances such as volatile anesthetics [2]. In different experimental studies, propofol-induced pre- and postconditioning demonstrated protective effects against I/R injury [61]. Underlying mechanisms include reduction in ROS and the regulation of Ca^2+^ overload through various channels (e.g., long lasting (L)- and transient opening (T)-type Ca^2+^ channels) during reperfusion. Emphasizing the most recent studies on this topic, a particular focus has been given on identifying the underlying mechanisms of cardioprotective effects by propofol in more detail.

Similar to the above-mentioned volatile anesthetics, recent studies revealed that regulation of miRNA expression seems to play a relevant role in terms of propofol-induced cardioprotection (Figure 1). Pretreatment with propofol in vivo and in vitro reduced infarct size and increased myocardial function by upregulation of miRNA-541 expression [62]. The respective miRNA in turn decreases expression of High Mobility Group Protein B1 (HMGB1), which is a mediator of apoptosis and has been shown to be involved in myocardial I/R injury. Furthermore, propofol regulates cardiac mast cells accumulating after myocardial infarction, possibly due to reduction of tryptase release, ultimately conferring cardioprotection [63,64]. Wang et al. investigated the influence of propofol on the transient receptor potential vanilloid (TRPV4) channel, especially focusing on changes of Ca^2+^ entry into cardiomyocytes. Propofol does attenuate I/R injury both in vitro and ex vitro and its cardioprotective action is—at least partially—mediated by the suppression of TRPV4 channel resulting in inhibition of intracellular Ca^2+^ overload [65]. Adverse effects induced by applying the TRPV4 agonist GSK1016790A were completely abolished by preconditioning with propofol in vitro. Moreover, propofol attenuates hypoxia/reperfusion (H/R)-induced intracellular Ca^2+^ overload ex vivo via the suppression of TRPV4 channel [65]. The involvement of TRPV4 underlying propofol’s cardioprotective effects might be an interesting starting point for future studies regarding pharmacological conditioning strategies.

Based on these data, propofol seems to be a promising agent in terms of pharmacological conditioning. However, as mentioned above, a combined administration of propofol with other pharmacological (or non-pharmacological) conditioning strategies seems to hamper cardioprotective effects. The exact mechanism of this paradox is still underexplored. One possible explanation may be that propofol has ROS scavenging abilities [61], which in turn might counteract the effects of other cardioprotective approaches. While cardioprotection by IPC is not attenuated by inducing anesthesia with propofol in an in vivo rat model of I/R [66], the protective effects of remote ischemic preconditioning (RIPC) are completely inhibited by the administration of propofol. This has been shown in an in vivo I/R rat model [67]. Interestingly, human plasma from RIPC-treated patients that received propofol for induction of general anesthesia forfeits its cardioprotective potential against I/R injury after transfer to isolated rat hearts [68]. These findings suggest that propofol may have an influence either on the target organ itself (in this case the heart) or on the release of humoral factors and their impact on I/R injury. In this context, Bunte et al. showed that only the release of humoral factors and not the direct effect on the heart were affected by propofol [66]. Transferring RIPC plasma from pentobarbital anesthetized rats to isolated rat hearts resulted in a reduced infarct size. In contrast to these findings, plasma collected from rats treated with RIPC undergoing propofol anesthesia did not show any cardioprotective effects in isolated hearts [66]. Interestingly, when administering plasma from RIPC-treated animals anesthetized with pentobarbital to propofol perfused isolated hearts mounted on a Langendorff system, cardioprotective properties of RIPC were fully effective. Comparable with these counteracting effects of propofol on RIPC, pharmacological-induced cardioprotection is also mitigated by propofol. Protective effects against myocardial I/R injury by preconditioning with phosphodiesterase inhibitors such as milrinone or levosimendan are fully abolished by propofol perfusion in an isolated rat heart I/R [69]. Similar results were observed in a working heart model by Lucchinetti et al. investigating postconditioning with Intralipid (ILPostC) [70]. Propofol perfusion as well as ILPostC alone, both sufficiently improved recovery of left ventricular work. However, the benefit of ILPostC was abolished in combination with propofol [70].

### 4.2. Clinical Implications

The contradictory behavior of propofol on I/R injury is crucial in the discussion why translation of cardioprotective strategies into the clinical setting so far has not been successful. ERICCA and RIPHEART—the two major clinical trials, being recalled when discussing failure of translation—were carried out in patients undergoing CABG surgery investigating the influence of RIPC on myocardial ischemia [71,72]. Notably, both studies used propofol-based anesthesia. Therefore, lack of protective effects of RIPC in these patients might be due to an abrogating effect of propofol. These hypotheses have been underlined by a secondary analysis of the RIPHEART trial [73]. This study revealed that RIPC did not affect the release of cardioprotective humoral factors and inflammatory biomarkers nor the activation of protein kinases involved in well-established signaling cascades [73]. Taking all these findings into account, it becomes apparent that more extensive research is needed to elucidate a possible negative impact of propofol in patients being investigated in the context of pharmacological conditioning strategies—especially in the clinical setting.

## 5. Opioids

### 5.1. Experimental Developments

Various review articles on cardioprotective effects of opioids have been published previously [74,75,76]. Regarding most recent literature, Melo et al., for instance, concentrated on non-coding RNAs and opioid-induced cardioprotection [77]. Non-coding RNAs, like miRNAs, have a profound influence on opioid receptors, regulate opioid signaling and are thus involved in the reduction of apoptosis and protection against postischemic myocardial damage [77]. Opioid receptors, e.g., kappa-opioid receptors (KOR), delta opioid receptors (DOR) and mu opioid receptors (MOR), are part of the G protein-coupled superfamily and next to the commonly known analgesic effect, they confer cardioprotection. While KOR and DOR are both expressed in the adult heart, cardiac expression of MOR depends on the species and its developmental stage. A multitude of signaling pathways in cardiomyocytes are known to be located downstream of and triggered by opioid receptors, finally inducing reduction in infarct size and protection against myocardial I/R injury [77].

Preconditioning with remifentanil confers cardioprotection against cardiac I/R injury comparable to IPC. This effect is meditated by the opioid receptors DOR and KOR as well as extracardiac MOR [76]. Notably, remifentanil-induced preconditioning has a second window of protection 24h after administration—comparable with IPC [76].

Interestingly, in healthy non-ischemic hearts, the MOR is practically absent, while these receptors are distinctively upregulated in the failing heart. This upregulation can be induced by the administration of doxorubicin or through myocardial infarction [78]. Naturally, this effect has a relevant impact on the underlying mechanism of protection by remifentanil. In the non-ischemic heart, infarct size reduction by treatment with remifentanil is completely abolished by the application of an antagonist that is selective for DOR and KOR. However, the administration of MOR-selective antagonists does not abolish cardioprotection. In contrast, under failing heart conditions opposite results are shown, where only the MOR antagonist blocks cardioprotection. It has to be mentioned that the myocardial MOR predominantly triggers opioid-induced conditioning via the ERK/glycogen synthase kinase 3 beta (GSK3β) signaling pathway [78]. In line with this, Jin et al. demonstrated that in the failing heart ERK and c-JUN N-terminal kinase (JNK) are both activated by remifentanil [78]. These MAPKs lead to a phosphorylation and consecutively inactivation of GSK3β. This is a major integration point of pro-survival protein kinases that results in regulation of apoptosis and protects against I/R injury [78]. The distinct role of GSK3β inhibition in protective effects of remifentanil is further underlined by a study of Chen et al. [79]. Remifentanil-induced postconditioning attenuates apoptosis in H9c2 cardiomyoblasts after I/R injury by inactivating GSK3β in a histone deacetylase 3 (HDAC3) dependent manner [79]. Moreover, Li et al. demonstrated that preconditioning with remifentanil has a dose-dependent cardioprotective effect, by improving myocardial dysfunction and reducing cell death after I/R injury [80].

Lastly, we want to emphasize the influence of different comorbidities on remifentanil-induced cardioprotection regarding translation into the clinical setting. It is recognized that both diabetes and acute hyperglycemia completely abolish the infarct size reduction by remifentanil. An explanation may be that increased oxidative stress leads to an impairment of caveolin-3 modulated PI3K/PKB and JAK2/STAT3 signaling [81].

### 5.2. Clinical Implications

Clinical evidence on cardioprotective effects of opioids is scarce. In cardiac surgery, commonly used opioids such as sufentanil or remifentanil reduce infarct size defined as decreased release of cardiac biomarkers. Aortic root infusion of sufentanil in patients undergoing mitral valve repair attenuated I/R injury as measured by significantly lower plasma concentrations of creatinine kinase (CK)-MB and troponin I [82]. In a small RCT (40 patients undergoing elective on-pump CABG surgery) the addition of remifentanil to the anesthesia regimen consisting of fentanyl and propofol reduced myocardial damage [83]. A meta-analysis including 1473 patients from 16 randomized trials stated that remifentanil reduced cardiac troponin release, duration of mechanical ventilation, and length of hospital stay in cardiac surgery patients [84].

As opioids are commonly used in cardiac surgery, the most interesting questions are: Which opioid is most protective and which dose is needed to achieve a cardioprotective effect? Both questions can currently not be answered, but available evidence suggests that cardioprotective doses are much higher than opioid doses routinely used for anesthesia [85]. Additional research is needed to finally define the role of cardioprotection by opioids in the clinical setting.

## 6. Alpha-2 Agonists

### 6.1. Experimental Developments

Two alpha-2 agonists have been investigated regarding their cardioprotective effects: Clonidine and dexmedetomidine. Application of the alpha-2 receptor agonist clonidine has long been suggested to improve outcome of high-risk cardiac patients undergoing surgery [86], most likely by blunting central sympathetic outflow. However, a direct conditioning effect of clonidine in human myocardial tissue has not been shown so far. As dexmedetomidine slowly replaces clonidine in the clinical setting, we will specifically highlight this agent in the following.

Dexmedetomidine is a highly selective alpha-2 receptor agonist that is clinically used for sedation or prevention/ therapy of postoperative delirium. It confers cardioprotection in pre, per- and postconditioning by mediating the reperfusion injury salvage kinase (RISK) pathway and activation of mitochondrial potassium channels (Figure 1) [87].

There are some distinct advantages of conditioning with dexmedetomidine over other substances: while for many other pharmacological agents, treatment immediately after the onset of reperfusion is necessary to achieve cardioprotection, for dexmedetomidine protection against I/R injury is completely independent of time and duration of application. In detail, 15 min of dexmedetomidine treatment that was initiated 45 min after ischemia was still effective in reducing infarct size [88]. These findings indicate a more extensive period for dexmedetomidine-induced cardioprotection after reperfusion which might give more flexibility for treatment in the clinical setting. Moreover, the protective effects of dexmedetomidine also seem to be maintained under pathological conditions. Cheng et al., demonstrated—in an in vivo rat model of I/R injury—that infarct size reduction by dexmedetomidine-induced postconditioning is not attenuated in type 2 diabetic rats in comparison with healthy rats [89]. In line with these results, our own research showed that preconditioning with dexmedetomidine confers cardioprotection despite the presence of acute hyperglycemia. Unfortunately, elevated glucose levels interfere with dexmedetomidine-induced postconditioning [90]. Additionally, in hearts with endothelial dysfunction, the protective effects of dexmedetomidine preconditioning are maintained [91]. In an isolated rat heart Langendorff system, endothelial dysfunction was induced by pretreatment with 60mM potassium and preconditioning with dexmedetomidine still induced protective effects on cell death and heart function after I/R injury [91]. These findings are comparable to IPC. Nonetheless, dexmedetomidine application is a noninvasive strategy which is a clear advantage. These mentioned aspects underline the promising potential of dexmedetomidine-induced cardioprotection in the clinical setting.

Besides the commonly known pathways in cardioprotective strategies, for dexmedetomidine—similar to other substances—the role of miRNA and protein expression was further investigated in recent years. Findings suggest that downregulation of miRNA-208 by dexmedetomidine alleviates apoptosis in cardiomyocytes [92]. A protective effect of dexmedetomidine by reducing apoptosis is not only mediated by miRNAs [92,93,94,95]—but also by hypoxia-inducible factor 1 alpha (Hif1α) signaling [96]. Peng et al. revealed that postconditioning with dexmedetomidine leads to a downregulation of Hif1α mRNA levels and its target gene BNIP3 [96]. Furthermore, apoptotic proteins such as cleaved caspase 3 and cleaved poly-ADP-ribose-polymerase 1 decreased after dexmedetomidine postconditioning. Overall, dexmedetomidine reduced apoptosis which consecutively resulted in protection against myocardial I/R injury in an in vivo rat model, as well as in ex vivo cardiomyocytes H/R experiments [96].

In accordance to dexmedetomidine-induced protection against apoptosis, Liu et al. demonstrated that treatment with dexmedetomidine likewise has an anti-apoptotic effect in H_2_O_2_-damaged neonatal rat cardiomyocytes by reducing oxidative stress in mitochondria and endoplasmic reticulum (ER) [97]. Focusing on the topic of protein expression in myocardial conditioning, treatment with dexmedetomidine seems to achieve its cardioprotective effects by enhancing the release of HMGB1 in an in vivo rat model of myocardial I/R [98]. This mechanism requires vagal nerve integrity and is dependent on alpha-7 nicotinic acetylcholine receptor (α7nAChR)-mediated cholinergic stimulation. Involvement of acetylcholine (ACh) receptor stimulation in protection against myocardial I/R has been described and reviewed extensively in the past [99]. However, these more recent findings suggest that cardioprotection may also be triggered by the cholinergic anti-inflammatory pathway and the activation of α7nAChR. Thus, the overexpression of HMGB1 may aggravate I/R injury or even abolish cardioprotection. Referring to this aspect, HMGB1 may be a key protein in myocardial conditioning by dexmedetomidine [98].

### 6.2. Clinical Implications

In the clinical setting, cardioprotective effects of alpha-2 agonists in humans might partly be a result of hemodynamic changes [100]. With regard to clonidine, a large RCT did not show a reduction of the composite outcome of death or nonfatal myocardial infarction in patients undergoing non-cardiac surgery (Table 1) [36]. Referring to dexmedetomidine, a small prospective trial including 38 patients undergoing CABG surgery indicated that myocardial damage was not reduced by dexmedetomidine, although a higher cardiac index and lower mean pulmonary arterial pressures were observed in the dexmedetomidine treated group [101]. In a retrospective analysis, Zhou et al. indicated that in patients undergoing valve surgery post-operative release of myocardial biomarkers (cardiac troponin I) was lower in patients receiving dexmedetomidine during the procedure [102], an observation that was confirmed in a small prospective randomized study in 28 patients undergoing valve replacement [103] and in patients undergoing CABG surgery [104]. However, large outcome trials are pending and the available data on clinically relevant cardioprotection by dexmedetomidine are of low to moderate quality.

## 7. Local Anesthetics

### 7.1. Experimental Developments

A further pharmacological agent that should be mentioned briefly is lidocaine. It is a routinely used local anesthetic and antiarrhythmic agent. Moreover, lidocaine confers cardioprotection after myocardial I/R injury [105,106]. As a cardiac sodium channel blocker, lidocaine ultimately leads to a reduction of intracellular Ca^2+^ levels [107]. In addition, lidocaine seems to reduce ROS production and modulates mitochondrial bioenergetics [108]. In the experimental setting, several animal studies revealed that systemic lidocaine is protective against myocardial I/R injury [109]. In the last two years, there are no relevant new findings according to our literature research. For more detailed general information on pharmacological conditioning with lidocaine we refer to published articles [110,111].

### 7.2. Clinical Implications

In the clinical setting, lidocaine could also show diverse beneficial effects which include reductions in pain, nausea, ileus duration, opioid use, and length of hospital stay [112]. Unfortunately, after CABG surgery studies have not shown any benefits in terms of postoperative pain treatment. However, Lee et al. did show in a RCT including 99 consecutive patients, that continuous i.v. application of lidocaine during surgery reduces myocardial injury in patients undergoing off-pump CABG surgery [113]. Wang et al. demonstrated that use of lidocaine results in a reduced rate of postoperative delirium after cardiac surgery. In this study, lidocaine was administered as a bolus of 1.5 mg/kg followed by a 4-mg/minute infusion added to CPB solution [114]. Another study by Mathew et al. also investigated this phenomenon, but could only show an effect for low doses of lidocaine in nondiabetic cardiac surgery patients [115]. In summary, the available clinical evidence currently is not sufficient to advice routine application of perioperative lidocaine infusion in cardiac patients.

## 8. Phosphodiesterase Inhibitors

### 8.1. Experimental Developments

Phosphodiesterase (PDE) inhibitors, such as milrinone (PDE3) and sildenafil (PDE5), have been investigated as possible agents of cardioprotection in several studies [116,117,118,119,120]. Hutschings et al. reviewed that PDE5 inhibitors effectively reduce infarct size and myocardial dysfunction [121]. Treatment with PDE3 inhibitors provides cardioprotective effects against I/R injury via activation of mitochondrial large-conductance calcium-sensitive potassium (mBK_Ca_) channels which finally results in suppression of mPTP (Figure 1) [116,117,118].

Noteworthy—and relevant for a possible translation into the clinical setting—, preconditioning effects of milrinone seem to depend on the anesthetic regimen: simultaneous perfusion with propofol or dexmedetomidine results in complete abrogation of cardioprotective properties by milirinone in isolated rat hearts [69]. This phenomenon was not found for simultaneous administration of sevoflurane.

The inodilator levosimendan—an agent with both positive inotropic and vasodilating effects—and its active metabolite OR-1896 also inhibit PDE3 [122]. Recently, the effect of levosimendan against doxorubicin-induced cardiotoxicity was reported [123]. An acute, single treatment with levosimendan reduced the detrimental effects of cardiotoxicity, e.g., myocardial dysfunction or oxidative stress, through PDE3 inhibition, resulting in an activation of the cAMP-PKA-PLN axis. This also led to a reduction of Ca^2+^ overload in cardiomyocytes [123]. Further studies did show that protection against I/R injury by levosimendan is meditated by mBK_Ca_, channels [124,125]. Interestingly, this agent forfeits its cardioprotective properties under simultaneous administration of propofol, but not under sevoflurane or dexmedetomidine anesthesia [69].

### 8.2. Clinical Implications

In clinical practice, inotropic agents such as milrinone improve cardiac function and have been shown to be beneficial in specific clinical situations, namely in cardiac surgery patients [126]. Clinical studies on cardioprotective effects of milrinone are not yet available.

## 9. Future Perspectives

The above cited data show a significant gap between experimental evidence and clinical effectiveness for perioperative cardioprotection. Various confounders and specific clinical circumstances have been suggested as underlying reasons [127]. These include age, presence of comorbidities, duration of disease and co-morbidity, co-medication for treatment of disease, acute treatment related to the intervention, use of anesthetic and analgesic drugs, as well as differences in measurement of end-points in experimental and various clinical settings [128].

Future studies should not only try to identify new cardioprotective agents, but also investigate a broader variety of cells—e.g., endothelium, neurons, etc.—and pathways—e.g., circulating cells, miRNA, mitochondrial receptor types—as possible targets of myocardial conditioning strategies. This seems especially relevant, as optimal clinical cardioprotection might need multiple interventions targeting different cell types and signaling pathways, as well as different time-points of treatment during I/R injury. Recently, multitarget strategies to reduce myocardial I/R injury have been formulated, looking for additive or synergistic cardioprotection from combined agents or interventions [129]. These could target the activation of pro-survival pathways (RISK, survivor activating factor enhancement (SAFE), protein kinase G) plus inhibition of cell death pathways and / or protection against different forms of cardiomyocyte death (necrosis, apoptosis, autophagy etc.) [130]. An example might be the protection by xenon (targeting signal transduction pathways) combined with hypothermia (targeting necrosis and apoptosis) [131]. Another strategy could be a combined cardiomyocyte and non-cardiomyocyte protection, e.g., improving coronary microcirculation by P2Y12-inhibitors. Co-medication might also enhance or restitute cardioprotection: statins have been shown to restore cardioprotection in diabetic animals [132], while other conditioning strategies seem to be less efficient in non-statin users [133]. Thus, optimal co-medications need to be defined to optimize perioperative cardioprotection.

Looking at currently planned or already recruiting clinical trials, there are some promising upcoming projects that might reveal important new information for the future. An example is the ProCCard trial (NCT03230136) which is a multicenter RCT investigating the effects of multimodal cardioprotection to reduce myocardial damage in patients undergoing cardiac surgery with CPB [134]. This trial combines five strategies of cardioprotection in the intervention group: (1) RIPC applied before aortic cross-clamping, (2) maintenance of anesthesia using sevoflurane, (3) tight intraoperative blood glucose management, (4) moderate respiratory acidosis at the end of CPB and (5) a gentle reperfusion protocol following aortic unclamping. The ProCCard trial already completed recruiting and might report first results in 2021. In our own working group, we are planning another multicenter RCT. This trial will investigate the effect of dexmedetomidine in cardiac surgery patients in terms of I/R injury and will also consider other potential cardioprotective factors, like influence of different anesthetic protocols.

## 10. Conclusions

To conclude, we have illustrated a variety of highly effective pharmacological approaches in protecting the heart against I/R injury in the experimental setting. However, translation into the clinical setting remains challenging. Some potential confounders have been identified that may contribute to the mainly negative results from previous clinical studies and at least some of them might be modifiable in the perioperative setting. With regard to future studies in this area, unraveling of the underlying cardiac but also extracardiac pathways should be a major focus of research. Moreover, carefully designed experimental and clinical studies evaluating combination of protective strategies targeting different cellular pathways, different cell types and different kinds of cell damage are warranted.

## Figures and Tables

**Table 1:** Important clinical trials on pharmacological conditioning.

Study Title	Population	Intervention	Endpoints	Results
Volatile anesthetics versus total intravenous anesthesia for cardiac surgery [34]	Elective CABG (RCT; *n* = 5400)	Volatile anesthetic vs. TIVA	Death from any cause at 1 year	No difference regarding deaths at 1 year between a volatile agent and total intravenous anesthesia
Volatile compared with total intravenous anaesthesia in patients undergoing high-risk cardiac surgery: a randomized multicentre study [33]	High-risk cardiac surgery patients with CPB (RCT; *n* = 200)	Sevoflurane versus TIVA	Composite of death, prolonged intensive care unit stay	No observed beneficial effect of sevoflurane on the composite endpoint
Sevoflurane Versus Total Intravenous Anesthesia for Isolated Coronary Artery Bypass Surgery with Cardiopulmonary Bypass: A Randomized Trial [32]	CABG with CPB (RCT; *n* = 868)	Sevoflurane versus TIVA	Hospital length of stay	Reduction of cardiac biomarker release and length of hospital stay after CABG by Sevoflurane
Randomized comparison of sevoflurane versus propofol to reduce perioperative myocardial ischemia in patients undergoing noncardiac surgery [35]	Noncardiac surgery patients at increased cardiovascular risk (RCT; *n* = 385)	Sevoflurane versus Propofol	Composite of myocardial ischemia detected by continuous ECG and/or troponin elevation	Sevoflurane did not reduce the incidence of myocardial ischemia
Clonidine in patients undergoing noncardiac surgery [36]	Patients at risk for atherosclerotic disease undergoing noncardiac surgery (RCT; *n* = 10,010)	Clonidine vs. Placebo	Composite endpoint of death or nonfatal myocardial infarction at 30 days	Clonidine did not reduce the rate of the composite outcome, but increased risk of hypotension and cardiac arrest
Effect of Xenon Anesthesia Compared to Sevoflurane and Total Intravenous Anesthesia for Coronary Artery Bypass Graft Surgery on Postoperative Cardiac Troponin Release [37]	Low-risk, on-pump CABG (RCT; *n* = 492)	Xenon vs. sevoflurane and TIVA	Cardiac troponin I concentration in the blood 24 h postsurgery	In postoperative troponin I release, xenon was noninferior to sevoflurane in CABG patients
Levosimendan in patients with left ventricular dysfunction undergoing cardiac surgery [38]	LVEF of 35% or less and cardiac surgery with CPB (RCT; *n* = 882)	Levosimendan vs. Placebo	Composite of death, RRT, MI and use of ECLS	Levosimendan did not reduce the incidence of the composite endpoint

RCT = randomized controlled trial; CABG = coronary artery bypass graft; TIVA = total intravenous anesthesia; LVEF = left ventricular ejection fraction; CPB = cardiopulmonary bypass; RRT = renal replacement therapy; MI = myocardial infarction; ECLS = extracorporeal life support; STEMI = ST-elevation myocardial infarction; MRI = magnetic resonance imaging.

## Data Availability

Not applicable.
